# Genome-wide analysis of zygotic linkage disequilibrium and its components in crossbred cattle

**DOI:** 10.1186/1471-2156-13-65

**Published:** 2012-07-24

**Authors:** Qi Jiang, Zhiquan Wang, Stephen S Moore, Rong-Cai Yang

**Affiliations:** 1 Department of Agricultural, Food and Nutritional Science, University of Alberta, Edmonton, AB, T6G 2P5, Canada; 2 Research and Innovation Division, Alberta Agriculture and Rural Development, Edmonton, Alberta, T6H 5T6, Canada

**Keywords:** Crossbred cattle, Gametic linkage disequilibrium, Genome-wide multilocus structure, Zygotic linkage disequilibrium

## Abstract

**Background:**

Linkage disequilibrium (LD) between genes at linked or independent loci can occur at gametic and zygotic levels known asgametic LD and zygotic LD, respectively. Gametic LD is well known for its roles in fine-scale mapping of quantitative trait loci, genomic selection and evolutionary inference. The less-well studied is the zygotic LD and its components that can be also estimated directly from the unphased SNPs.

**Results:**

This study was set up to investigate the genome-wide extent and patterns of zygotic LD and its components in a crossbred cattle population using the genomic data from the Illumina BovineSNP50 beadchip. The animal population arose from repeated crossbreeding of multiple breeds and selection for growth and cow reproduction. The study showed that similar genomic structures in gametic and zygotic LD were observed, with zygotic LD decaying faster than gametic LD over marker distance. The trigenic and quadrigenic disequilibria were generally two- to three-fold smaller than the usual digenic disequilibria (gametic or composite LD). There was less power of testing for these high-order genic disequilibria than for the digenic disequilibria. The power estimates decreased with the marker distance between markers though the decay trend is more obvious for the digenic disequilibria than for high-order disequilibria.

**Conclusions:**

This study is the first major genome-wide survey of all non-allelic associations between pairs of SNPs in a cattle population. Such analysis allows us to assess the relative importance of gametic LD vs. all other non-allelic genic LDs regardless of whether or not the population is in HWE. The observed predominance of digenic LD (gametic or composite LD) coupled with insignificant high-order trigenic and quadrigenic disequilibria supports the current intensive focus on the use of high-density SNP markers for genome-wide association studies and genomic selection activities in the cattle population.

## Background

The recent advancement in molecular biology has enabled the rapid development of single nucleotide polymorphism (SNP) genotyping technology, thereby making the genotyping of cheap and abundant SNP markers possible in many livestock species [1]. Commercial SNP chips (e.g., Illumina BovineSNP50 beadchip for cattle) are now available for high-throughput genotyping of large numbers of SNPs in cattle, which allows animal geneticists to search for the quantitative trait nucleotides (QTNs) underlying variation in complex traits from genome-wide association studies (GWAS) or to predict animal’s performance through genomic selection [2,3]. The success of GWAS and genomic selection depends crucially on the extent of linkage disequilibrium (LD) between SNPs and QTNs on chromosomes. It is shown (e.g., [3]) that strong gametic LD is found at long distances (> 1 cM) in domestic animals (e.g., cattle and dog) but not in human.

With the availability of high-density SNPs, many studies have also conducted population genetic analysis of gametic LD in cattle and other animal species (e.g., [4-8]). However, the gametic LD cannot be calculated directly for unphased SNP markers because the gametic phase of animals that are heterozygous at two or more loci cannot be directly observed or specified. Thus, these studies have often followed the classic approach of Hill [9] to estimating gametic LD for unphased data, but such estimation was carried out under the assumption of Hardy-Weinberg equilibrium (HWE). For pure breed populations as in the above studies, the HWE assumption may be reasonable.

In this study, we investigate the genome-wide extent and patterns of LD in a crossbred cattle population using the genomic data typed with the Illumina BovineSNP50 beadchip. The animals in this study are the progenies of three synthetic lines that were maintained separately at the University of Alberta Kinsella Research Ranch during 1960 to 1989 [10] and were subsequently pooled. It will hereafter be called as the Kinsella composite beef population. Since this population arose from repeated mixing of multiple breeds and selection for growth and cow reproduction, it may not be in HWE. Furthermore, in general diploid nonequilibrium populations such as the Kinsella composite beef population, Yang [11,12] suggested the assessment of LD at both gametic and zygotic levels. Higher-order trigenic and quadrigenic disequilibria are the components of the zygotic LD. To the best of our knowledge, only one study by Liu et al. [13] examined the trigenic and quadrigenic disequilibria in a canine population but it was based on a small number of dogs and a limited number of markers. Additionally, all LD measures (zygotic LD and its components) are known to depend on gene frequencies at different loci, but such dependence is rarely examined. For example, gametic LD is generally smaller if gene frequencies are near fixation than if gene frequencies are intermediate. Thus there is a need to examine the dependence of zygotic LD and its components on gene frequencies.

The Kinsella beef composite population has been the subject of numerous breeding and genomic studies (e.g., [14-17]). However, the extent and patterns of gametic LD or other LD measures in this population has never been examined. It would be desirable to fully investigate the extent of LD and their distribution patterns of the whole genome for this population to provide the baseline information about multilocus structures for helping future genomic research. Therefore, the objectives of this study are (i) to determine if individual genic disequilibria are significant; (ii) to investigate the relationship between individual components of zygotic LD and physical distance; and (iii) to examine effects of changing gene frequencies on different LD measures.

## Results

### Zygotic LD and its components

**Figure 1:**
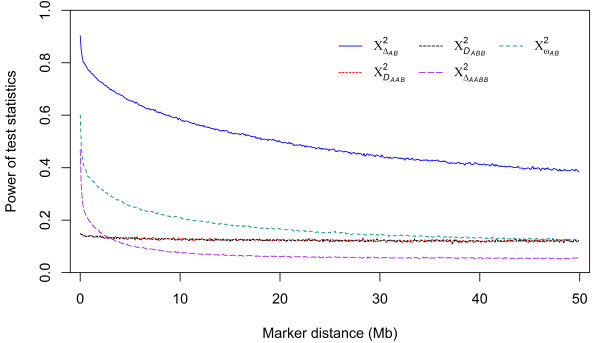
**The relationship between the estimated powers of chi-square tests for zygotic LD and its individual genic components and marker distance for SNP markers that were apart within 50 Mb on 29 autosomes in the Kinsella beef composite population.** Note that the powers of the tests for the two trigenic components were very similar over the whole range of marker distance as indicated by the lines for the two disequilibria being overlapped to each other.

The estimates of power of chi-square tests for zygotic LD and individual genic disequilibria were plotted against the marker distances of ≤ 50 Mb for all SNP pairs on 29 autosomes (Figure 1). This plot showed that the power decreased with the increasing marker distance. The pace of the power decay varied with individual genic disequilibria with the composite LD being the slowest but the two trigenic disequilibria being the fastest. After this initial sharp decline, the power values were stabilized around 0.4 for the composite LD, 0.2 for the two trigenic disequilibria and 0.05 for the quadrigenic disequilibria over the range of ≤ 50 Mb and beyond. Since the most useful LDs occurred for SNP pairs spanning at close vicinity (≤ 5 Mb) as shown in Figure 1, we calculated gametic, composite and zygotic LDs in terms of squared correlation at nine distance intervals within 5 Mb for all SNP pairs within individual distance intervals and the percentages of LD values being ≥0.25 (Table 1). There was a clear trend of LD decay with the marker distance. The trigenic and quadrigenic disequilibria were not shown because there was little trend of decay for these disequilibria even within 5 Mb.

**Table 1:** **Means and standard deviations (SD) of gametic LD (**${\varphi_{\mathrm{GLD}}^{2}}^{a}$**), composite LD (**$\varphi_{\mathrm{CLD}}^{2}$**) and zygotic LD (**${\varphi_{\mathrm{ZLD}}^{2}}^{a}$**) between syntenic SNP pairs for different ranges of distance (≤ 5 Mb) in the Kinsella composite beef population**

**Distance range (Mb)**	**Pairs (n)**	$\varphi_{\mathrm{GLD}}^{2}$**±SD**	$\varphi_{\mathrm{CLD}}^{2}$**± SD**	$\varphi_{\mathrm{ZLD}}^{2}$**± SD**	$\varphi_{\mathrm{GLD}}^{2}$**≥0.25 (%)**	$\varphi_{\mathrm{CLD}}^{2}$**≥0.25 (%)**	$\varphi_{\mathrm{ZLD}}^{2}$**≥0.25 (%)**
<0.025	5692	0.2853±0.3071	0.2857±0.3070	0.1812±0.3011	38.3	38.5	22.9
0.025-0.05	20032	0.2175±0.2638	0.2178±0.2638	0.1230±0.2425	29.5	29.4	15.8
0.05-0.075	18041	0.1615±0.2191	0.1619±0.2192	0.0799±0.1884	20.8	20.9	10.0
0.075-0.1	17838	0.1277±0.1832	0.1278±0.1832	0.0561±0.1479	15.6	15.5	6.7
0.1-0.2	69905	0.0912±0.1420	0.0914±0.1422	0.0340±0.1059	9.4	9.5	3.6
0.2-0.5	205321	0.0569±0.0885	0.0571±0.0887	0.0159±0.0559	3.7	3.7	1.0
0.5-1.5	665397	0.0394±0.0580	0.0395±0.0581	0.0091±0.0300	1.3	1.3	0.3
1.5-3	968768	0.0286±0.0424	0.0286±0.0424	0.0062±0.0195	0.5	0.5	0.1
3-5	1252994	0.0214±0.0315	0.0214±0.0316	0.0045±0.0131	0.1	0.1	0.0

Percentages of SNP pairs with LD values of ≥0.25 are also presented. ^a^ The Generalized measures of gametic and zygotic LD ($\varphi_{\mathrm{GLD}}^{2}$ and $\varphi_{\mathrm{ZLD}}^{2}$) are based on an exact 2 × 2 contingency table and thus they are simply the squared correlations, i.e., $\varphi_{\mathrm{GLD}}^{2}=r_{\mathrm{GLD}}^{2}$ and $\varphi_{\mathrm{ZLD}}^{2}=r_{\mathrm{ZLD}}^{2}$.

Similar trend of LD decay was observed on individual chromosomes. However, given the limited numbers of SNP pairs over the nine intervals within the short distance of ≤ 5 Mb on individual chromosomes, the estimates of power of chi-square test statistic for gametic, composite and zygotic LDs are presented for two groups of SNP pairs, those with a distance of ≤ 50 Mb (Linked Group) and those with a distance of > 50 Mb (Unlinked Group) (Table 2). As in the usual convention, any marker pair spanning > 50 Mb would be considered freely recombined or unlinked, assuming the one-to-one relationship between the genetic and physical distances (i.e., 1cM = 1 Mb). The physical distances for individual chromosomes are listed in Table A 1. Within each group, the chi-square tests for gametic LD and composite LD had similar power, but they both had higher values than the chi-square tests for zygotic LD. Genome-wide, the chi-square tests for gametic and composite LD were ~15% more powerful in the Linked Group than in the Unlinked Group, but the chi-square tests for zygotic LD were only ~7% more powerful in the Linked Group than in the Unlinked Group. For the Linked Group, the range of mean estimates of power for gametic LD was from 50.6% on BTA 19 to 53.9% on BTA 25; the range of mean estimates of power for composite LD was from 50.6% on BTA 19 to 54.0% on BTA 25; and the range of mean estimates of power for zygotic LD was from 16.9% on BTA 24 to 19.7% on BTA 11. The corresponding ranges of mean estimates of power for the Unlinked Group were 0.348 (BTA12) – 0.401 (BTA 29) for gametic LD, 0.344 (BTA 12) – 0.392 (BTA 29) for composite LD and 0.105 (BTA 23) – 0.126 (BTA 11) for zygotic LD. It should be noted that chromosomes 25, 27 and 28 are shorter than 50 Mb, and chromosome 26 is 52 Mb long but the chi-square statistics for all marker pairs with a distance >50 Mb did not exceed 3.84.

**Table 2:** The power values^*^of test statistics for gametic LD, composite LD and zygotic LD for marker pairs with a distance ≤50 Mb and >50 Mb on 29 autosomes in the Kinsella composite beef population

**BTA**	$X_{D_{\mathrm{AB}}}^{2}$		$X_{\Delta_{\mathrm{AB}}}^{2}$		$X_{\omega_{\mathrm{AB}}}^{2}$	
	**≤50 Mb**	**>50 Mb**	**≤50 Mb**	**>50 Mb**	**≤50 Mb**	**>50 Mb**
1	0.522	0.358	0.525	0.359	0.193	0.119
2	0.536	0.374	0.536	0.372	0.189	0.119
3	0.524	0.364	0.523	0.358	0.193	0.122
4	0.531	0.379	0.531	0.378	0.190	0.120
5	0.531	0.375	0.532	0.373	0.194	0.124
6	0.527	0.359	0.524	0.353	0.191	0.115
7	0.517	0.375	0.515	0.371	0.189	0.119
8	0.528	0.374	0.530	0.372	0.192	0.121
9	0.511	0.362	0.512	0.361	0.188	0.122
10	0.535	0.373	0.538	0.372	0.197	0.125
11	0.529	0.387	0.529	0.385	0.189	0.126
12	0.513	0.348	0.514	0.344	0.182	0.114
13	0.517	0.352	0.515	0.349	0.185	0.114
14	0.524	0.375	0.522	0.370	0.177	0.112
15	0.521	0.367	0.521	0.364	0.191	0.121
16	0.511	0.365	0.514	0.369	0.184	0.121
17	0.518	0.360	0.519	0.361	0.191	0.120
18	0.517	0.386	0.514	0.383	0.172	0.107
19	0.506	0.368	0.506	0.369	0.169	0.116
20	0.530	0.378	0.531	0.372	0.191	0.118
21	0.508	0.375	0.507	0.372	0.182	0.119
22	0.517	0.374	0.518	0.369	0.189	0.123
23	0.513	0.359	0.510	0.347	0.169	0.105
24	0.536	0.376	0.535	0.372	0.188	0.113
25	0.539	-^a^	0.540	-	0.170	-
26	0.518	0.000^b^	0.530	0.000	0.170	0.000
27	0.537	-	0.538	-	0.189	-
28	0.521	-	0.518	-	0.186	-
29	0.531	0.401	0.531	0.392	0.185	0.123
Overall	0.523	0.371	0.523	0.367	0.185	0.118

* Proportion of marker pairs that *X*^2^ exceeded 3.84, the 5% critical value of $\chi_{\left(1\right)}^{2}$.

^a^ The chromosome length was shorter than 50 Mb and there were no marker pairs with a distance that would exceed 50 Mb.

^b^ Chromosome 26 was 52 Mb long but no marker pairs with a distance of >50 Mb had a chi-square statistic that exceeded 3.84.

The estimates of power of the chi-square tests for trigenic and quadrigenic components of the zygotic LD are given for the Linked (≤ 50 Mb) and Unlinked (> 50 Mb) Groups (Table 3). For a given trigenic or quadrigenic disequilibrium, the power estimates of chi-square tests were similar regardless of the distance between marker pairs. The ranges of the power for each of the two trigenic disequilibria were 0.103-0.145 in the Linked Group and 0.081-0.184 in the Unlinked Group. Such ranges for the quadrigenic disequilibrium were 0.072-0.092 in the Linked Group and 0.052-0.066 in the Unlinked Group. It should be noted that the estimates of power were based on the number of marker pairs left after removing those with the generalized squared correlations ($\varphi^{2}$) being outside the acceptable range of 0 to 1. We recorded separately the frequencies of the two out-of-bound situations ($\varphi^{2}$< 0 and $\varphi^{2}$ > 1) in Table A2. First, for $\varphi^{2}$ < 0, the sampling variances of estimated trigenic disequilibria were negative for about 69% of the genome-wide syntenic marker pairs (36,131,636); in contrast, the sampling variances of estimated quadrigenic disequilibrium were positive for all the syntenic marker pairs. Second, for $\varphi^{2}$ > 1, there was 0.02% of the genome-wide syntenic marker pairs for both trigenic disequilibria, but only 0.001% for quadrigenic disequilibrium.

**Table 3:** The power estimates* of test statistics for the trigenic and quadrigenic disequilibria for marker pairs with a distance ≤50 Mb and >50 Mb on 29 autosomes in the Kinsella composite beef population

**BTA**	$X_{D_{\mathrm{ABB}}}^{2}$		$X_{D_{\mathrm{AAB}}}^{2}$		$X_{\Delta_{\mathrm{ABAB}}}^{2}$	
	**≤50 Mb**	**>50 Mb**	**≤50 Mb**	**>50 Mb**	**≤50 Mb**	**>50 Mb**
1	0.124	0.122	0.120	0.108	0.076	0.052
2	0.140	0.130	0.140	0.134	0.077	0.054
3	0.119	0.113	0.120	0.115	0.076	0.053
4	0.119	0.119	0.117	0.115	0.082	0.055
5	0.131	0.126	0.133	0.124	0.079	0.053
6	0.124	0.120	0.121	0.108	0.079	0.052
7	0.114	0.114	0.115	0.121	0.078	0.056
8	0.122	0.119	0.119	0.114	0.081	0.056
9	0.128	0.119	0.135	0.140	0.072	0.053
10	0.129	0.126	0.126	0.118	0.082	0.055
11	0.132	0.126	0.131	0.129	0.078	0.053
12	0.128	0.120	0.125	0.118	0.076	0.053
13	0.122	0.127	0.122	0.117	0.082	0.055
14	0.120	0.113	0.126	0.122	0.084	0.053
15	0.128	0.137	0.127	0.138	0.075	0.056
16	0.135	0.097	0.135	0.120	0.073	0.053
17	0.103	0.111	0.112	0.134	0.076	0.052
18	0.145	0.165	0.131	0.138	0.078	0.055
19	0.126	0.178	0.117	0.116	0.078	0.053
20	0.134	0.126	0.136	0.133	0.077	0.055
21	0.127	0.116	0.117	0.092	0.072	0.052
22	0.114	0.081	0.127	0.148	0.079	0.055
23	0.141	0.184	0.129	0.117	0.08	0.059
24	0.139	0.116	0.145	0.137	0.083	0.055
25	0.145	-^a^	0.142	-	0.092	-
26	0.115	0.000^b^	0.110	0.000	0.077	0.000
27	0.12	-	0.126	-	0.078	-
28	0.122	-	0.138	-	0.077	-
29	0.141	0.121	0.125	0.107	0.077	0.066
Overall	0.130	0.125	0.130	0.123	0.080	0.055

* Proportion of marker pairs that *X*^2^ exceeded 3.84, the 5% critical value of $\chi_{\left(1\right)}^{2}$.

^a^ The chromosome length was shorter than 50 Mb and there were no marker pairs with a distance that would exceed 50 Mb.

^b^ Chromosome 26 was 52 Mb long but no marker pairs with a distance of >50 Mb had a chi-square statistic that exceeded 3.84.

Presented in Table 4 are the generalized squared correlations ($\varphi^{2}$) of gametic, composite, trigenic and quadrigenic disequilibria averaged over all syntenic marker pairs. The genome-wide $\varphi^{2}$values for digenic disequilibria (gametic and composite LD) were about three times those of trigenic and quadrigenic disequilibria. There was variation in individual genic disequilibria among chromosomes. For example, the gametic LD averaged over all pairs on chromosomes ranged from 0.0082 on BTA 1 to 0.0126 on BTA 25 whereas the quadrigenic LD ranged from 0.0012 on BTA1 to 0.0016 on BTA 25. When looking at the percentages of SNP pairs with $\varphi^{2}$ ≥ 0.2 (Table 5), the values for digenic disequilibria were also two to three times those for trigenic and quadrigenic disequilibria.

**Table 4:** The estimated digenic (gametic and composite), trigenic and quadrigenic disequilibria averaged over all syntenic SNP pairs on 29 autosomes in the Kinsella composite beef population

**BTA**	$\varphi_{D_{\mathrm{AB}}}^{2}{}^{a}$	$\varphi_{\Delta_{\mathrm{AB}}}^{2}{}^{a}$	$\varphi_{D_{\mathrm{ABB}}}^{2}{}^{a}$	$\varphi_{D_{\mathrm{ABB}}}^{2}{}^{a}$	$\varphi_{\Delta_{\mathrm{ABAB}}}^{2}{}^{a}$
1	0.0082	0.0077	0.0035	0.0033	0.0012
2	0.0088	0.0082	0.0036	0.0036	0.0012
3	0.0093	0.0085	0.0030	0.0032	0.0013
4	0.0099	0.0091	0.0034	0.0032	0.0013
5	0.0096	0.0089	0.0037	0.0037	0.0013
6	0.0095	0.0086	0.0031	0.0032	0.0013
7	0.0098	0.0089	0.0031	0.0032	0.0013
8	0.0098	0.0090	0.0033	0.0033	0.0013
9	0.0092	0.0085	0.0036	0.0036	0.0012
10	0.0101	0.0094	0.0034	0.0031	0.0013
11	0.0098	0.0090	0.0036	0.0039	0.0013
12	0.0099	0.0091	0.0036	0.0034	0.0013
13	0.0109	0.0099	0.0034	0.0032	0.0014
14	0.0111	0.0101	0.0031	0.0035	0.0014
15	0.0103	0.0096	0.0033	0.0038	0.0013
16	0.0103	0.0095	0.0041	0.0040	0.0013
17	0.0103	0.0096	0.0032	0.0031	0.0013
18	0.0111	0.0103	0.0039	0.0034	0.0014
19	0.0108	0.0100	0.0034	0.0032	0.0014
20	0.0108	0.0100	0.0037	0.0037	0.0013
21	0.0103	0.0095	0.0035	0.0030	0.0013
22	0.0113	0.0104	0.0028	0.0032	0.0014
23	0.0111	0.0102	0.0045	0.0036	0.0014
24	0.0123	0.0112	0.0039	0.0041	0.0014
25	0.0126	0.0117	0.0044	0.0037	0.0016
26	0.0118	0.0111	0.0032	0.0031	0.0014
27	0.0124	0.0114	0.0035	0.0035	0.0014
28	0.0113	0.0104	0.0033	0.0036	0.0014
29	0.0115	0.0106	0.0041	0.0035	0.0014
Ave.	0.0105	0.0097	0.0035	0.0034	0.0013

^a^ The generalized measures of squared correlation for digenic, trigenic and quadrigenic disequilibria:

$\varphi_{D_{\mathrm{AB}}}^{2}=X_{D_{\mathrm{AB}}}^{2}/n\text{,}\;\varphi_{\Delta_{\mathrm{AB}}}^{2}=X_{\Delta_{\mathrm{AB}}}^{2}/n\text{,}\;\varphi_{D_{\mathrm{ABB}}}^{2}=X_{D_{\mathrm{ABB}}}^{2}/n\text{,}\;\varphi_{D_{\mathrm{AAB}}}^{2}=X_{D_{\mathrm{AAB}}}^{2}=X_{D_{\mathrm{AAB}}}^{2}/n\text{,}\;and\;\varphi_{\Delta_{\mathrm{AABB}}}^{2}=X_{\Delta_{\mathrm{AABB}}}^{2}/n$, where *n* is the number of animals at individual SNP pairs.

**Table 5:** The proportion of syntenic SNP pairs with the generalized measures of square correlation estimated for gametic, composite, trigenic and quadrigenic disequilibria that exceeded 0.2 on 29 autosomes in the Kinsella composite beef population

**BTA**	$\varphi_{D_{{}^{\mathrm{AB}}}}^{2}{}^{a}$	$\varphi_{D_{\mathrm{AB}}}^{2}{}^{a}$	$\varphi_{D_{\mathrm{ABB}}}^{2}$	$\varphi_{D_{\mathrm{ABB}}}^{2}$	$\varphi_{\Delta }^{2}{}_{{}_{\mathrm{ABAB}}}$
1	0.1366	0.1440	0.0711	0.0666	0.0050
2	0.1345	0.1279	0.0626	0.0619	0.0061
3	0.1971	0.1778	0.0487	0.0559	0.0068
4	0.2368	0.2326	0.0676	0.0672	0.0109
5	0.2212	0.2132	0.0745	0.0775	0.0077
6	0.1934	0.1690	0.0615	0.0664	0.0074
7	0.2418	0.2298	0.0650	0.0617	0.0104
8	0.2230	0.2137	0.0559	0.0622	0.0105
9	0.1744	0.1692	0.0681	0.0646	0.0081
10	0.2147	0.2162	0.0623	0.0476	0.0074
11	0.2066	0.1984	0.0770	0.0816	0.0084
12	0.1782	0.1768	0.0744	0.0669	0.0081
13	0.2926	0.2607	0.0620	0.0584	0.0113
14	0.2836	0.2851	0.0645	0.0750	0.0080
15	0.2052	0.1972	0.0587	0.0804	0.0056
16	0.2507	0.2466	0.0834	0.0850	0.0130
17	0.2079	0.2109	0.0738	0.0600	0.0075
18	0.2494	0.2349	0.0760	0.0596	0.0094
19	0.2417	0.2262	0.0610	0.0655	0.0102
20	0.2130	0.2055	0.0759	0.0681	0.0073
21	0.2108	0.1957	0.0726	0.0589	0.0074
22	0.2943	0.3005	0.0462	0.0475	0.0085
23	0.2408	0.2230	0.1060	0.0704	0.0131
24	0.3267	0.2822	0.0795	0.0911	0.0099
25	0.3060	0.3160	0.1130	0.0848	0.0155
26	0.2806	0.2806	0.0623	0.0635	0.0108
27	0.2947	0.2962	0.0746	0.0725	0.0091
28	0.2213	0.2096	0.0497	0.0569	0.0075
29	0.2396	0.2358	0.0849	0.0756	0.0103
Ave.	0.2316	0.2233	0.0701	0.0674	0.0090

^a^ The generalized measures of squared correlation for digenic, trigenic and quadrigenic disequilibria:

$\varphi_{D_{\mathrm{AB}}}^{2}=X_{D_{\mathrm{AB}}}^{2}/n\text{,}\;\varphi_{\Delta_{\mathrm{AB}}}^{2}=X_{\Delta_{\mathrm{AB}}}^{2}/n\text{,}\;\varphi_{D_{\mathrm{ABB}}}^{2}=X_{D_{\mathrm{ABB}}}^{2}/n\text{,}\;\varphi_{D_{\mathrm{AAB}}}^{2}=X_{D_{\mathrm{AAB}}}^{2}=X_{D_{\mathrm{AAB}}}^{2}/n\text{,}\;and\;\varphi_{\Delta_{\mathrm{AABB}}}^{2}=X_{\Delta_{\mathrm{AABB}}}^{2}/n$, where *n* is the number of animals at individual SNP pairs.

The mean values of LDs and power estimates for chi-square tests for gametic, composite, trigenic, quadrigenic and zygotic LD were summarized for all syntenic marker pairs (intra-chromosome pairs) and all non-syntenic pairs (inter-chromosome pairs) (Table 6). The mean values of individual genic disequilibria and test power were greater for syntenic marker pairs than for non-syntenic marker pairs though such difference between syntenic- vs. non-syntenic pairs were more pronounced for the digenic disequilibria than for trigenic and quadrigenic disequilibria. In particular, the two trigenic disequilibria and their test power were almost the same for syntenic and non-syntenic pairs. The magnitudes of LD values and test powers decreased with the number of alleles in the LD measures for both syntenic and non-syntenic marker pairs with the order of digenic LD > trigenic LD > quadrigenic LD. It should be noted that despite the same number of possible intra- or inter-chromosome marker pairs for all individual genic disequilibria, only those pairs whose generalized squared correlations fell within the acceptable range of 0 ≤ $\varphi^{2}$≤ 1 were retained for calculating the mean LD values and estimating the test powers.

**Table 6:** Means and ranges of generalized measures of squared correlations for digenic (gametic and composite), trigenic, quadrigenic and zygotic disequilibria averaged over all syntenic (intra-chromosome) SNP pairs and over all non-syntenic (inter-chromosome) SNP pairs across the composite beef genome

**LD**	**Intra-chromosome**							**Inter-chromosome**				
	**# of pairs**	**Strength**			Power^*^			**# of pairs**	**Strength**		Power^*^	
		**Mean**	**Range**		**Mean**	**Range**			**Mean**	**Range**	**Mean**	**Range**
$\varphi_{D_{\mathrm{AB}}}^{2}{\left(r_{D_{\mathrm{AB}}}^{2}\right)}^{\text{a}}$	36131611	0.0105	0.0082-0.0126		0.4946	0.4432-0.5385		83686490	0.0044	0.0041-0.0047	0.3526	0.3338-0.3740
$\varphi_{\Delta_{\mathrm{AB}}}^{2}{}^{a}$	36131636	0.0097	0.0077-0.0117		0.4945	0.4449-0.5400		893686490	0.0044	0.0041-0.0047	0.3526	0.3338-0.3740
$\varphi_{D_{\mathrm{ABB}}}^{2}{}^{\text{a}}$	11158132	0.0035	0.0028-0.0045		0.1264	0.1043-0.1459		272879855	0.0033	0.0027-0.0042	0.1198	0.1025-0.1404
$\varphi_{D_{\mathrm{AAB}}}^{2}{}^{\text{a}}$	11224541	0.0034	0.0030-0.0041		0.1257	0.1098-0.1450		273790442	0.0034	0.0027-0.0042	0.1222	0.1030-0.1428
$\varphi_{\Delta_{\mathrm{AABB}}}^{2}{}^{\text{a}}$	36128429	0.0013	0.0012-0.0016		0.0740	0.0643-0.0921		893685393	0.0010	0.0010-0.0011	0.0540	0.0513-0.0579
$\varphi_{\omega_{\mathrm{AB}}}^{2}{\left(r_{\omega \mathrm{AB}}^{2}\right)}^{\text{a}}$	36071428	0.0029	0.0025-0.0034		0.1726	0.1577-0.1887		893012960	0.0016	0.0015-0.0016	0.1145	0.0105-0.1205

* Proportion of SNP pairs that *X*^2^ exceeded 3.84, the 5% critical value of $\chi_{\left(1\right)}^{2}$.

^a^ The generalized measures of squared correlation for digenic, trigenic and quadrigenic disequilibria: $\varphi_{D_{\mathrm{AB}}}^{2}=X_{D_{\mathrm{AB}}}^{2}/n\text{,}\;\varphi_{\Delta_{\mathrm{AB}}}^{2}=X_{\Delta_{\mathrm{AB}}}^{2}/n\text{,}\;\varphi_{D_{\mathrm{ABB}}}^{2}=X_{D_{\mathrm{ABB}}}^{2}/n\text{,}\;\varphi_{D_{\mathrm{AAB}}}^{2}=X_{D_{\mathrm{ABB}}}^{2}/n\text{,}\;\varphi_{\Delta_{\mathrm{AABB}}}^{2}=X_{\Delta_{\mathrm{AABB}}}^{2}/n\;and\;\varphi_{\omega_{\mathrm{AB}}}^{2}=X_{\omega_{\mathrm{AB}}}^{2}/n$, where *n* is the number of animals at individual SNP pairs. The Generalized measures of gametic and zygotic LD ($\varphi_{\mathrm{GLD}}^{2}$ and $\varphi_{\mathrm{ZLD}}^{2}$) are based on an exact 2 × 2 contingency table and thus they are simply the squared correlations, i.e., $\varphi_{\mathrm{GLD}}^{2}=r_{\mathrm{GLD}}^{2}$ and $\varphi_{\mathrm{ZLD}}^{2}=r_{\mathrm{ZLD}}^{2}$.

### Dependence of LD on gene frequency

The power of chi-square tests for individual genic disequilibria was estimated for syntenic marker pairs belonging to nine classes of minor allele frequency (MAF) with three MAF intervals (< 0.1, 0.1-0.3 and 0.3-0.5) at each of the two loci on each of 29 chromosomes as well as for non-syntenic marker pairs on two different chromosomes. The power estimates for non-syntenic marker pairs were calculated on all 406 [29(29-1)/2] chromosome pairs. In Table 7 are given the mean, minimum, and maximum values of power estimates for the syntenic marker pairs over 29 chromosomes and for non-syntenic pairs over 406 chromosome pairs. In all nine MAF classes, the digenic disequilibria (composite LD) and zygotic LD were greater for intra-chromosome pairs than for inter-chromosome pairs but trigenic and quadrigenic disequilibria were similar for both intra- and inter-chromosome pairs.

**Table 7:** The mean, minimum and maximum of power estimates^*^of the test statistics for digenic, trigenic and quadrigenic disequilibria obtained for nine combinations of minor allele frequency (MAF) categories at each of the two loci (MAF_**A**_**and MAF**_**B**_**) for all syntenic (intra-chromosome) SNP pairs over 29 chromosomes and for all non-syntenic (inter-chromosome) SNP pairs over 406 [29(29-1)/2] chromosome pairs in the Kinsella composite beef population**

**MAF**_**A**_	**MAF**_**B**_		**Intra-chromosome**					**Inter-chromosome**				
			$X_{\Delta_{\mathrm{AB}}}^{2}$	$X_{D_{\mathrm{ABB}}}^{2}$	$X_{D_{\mathrm{AAB}}}^{2}$	$X_{\Delta_{\mathrm{AABB}}}^{2}$	$X_{\omega_{\mathrm{AB}}}^{2}$	$X_{\Delta_{\mathrm{AB}}}^{2}$	$X_{D_{\mathrm{ABB}}}^{2}$	$X_{D_{\mathrm{AAB}}}^{2}$	$X_{\Delta_{\mathrm{AABB}}}^{2}$	$X_{\omega_{\mathrm{AB}}}^{2}$
	< 0.1	Mean	0.389	0.031	0.032	0.018	0.290	0.282	0.014	0.014	0.022	0.212
< 0.1		Min	0.342	0.016	0.008	0.013	0.251	0.243	0.002	0.004	0.017	0.183
		Max	0.444	0.049	0.084	0.024	0.328	0.317	0.037	0.052	0.027	0.241
												
	0.1-0.3	Mean	0.464	0.000	0.056	0.029	0.308	0.323	0.000	0.035	0.029	0.202
		Min	0.396	0.000	0.034	0.024	0.261	0.286	0.000	0.022	0.026	0.180
		Max	0.544	0.002	0.104	0.036	0.364	0.361	0.000	0.058	0.034	0.225
												
	0.3-0.5	Mean	0.477	0.000	0.187	0.051	0.136	0.323	0.000	0.183	0.041	0.094
		Min	0.420	0.000	0.157	0.039	0.120	0.286	0.000	0.154	0.034	0.086
		Max	0.565	0.000	0.216	0.072	0.160	0.361	0.000	0.219	0.049	0.104
												
	< 0.1	Mean	0.463	0.053	0.000	0.030	0.310	0.320	0.034	0.000	0.030	0.204
0.1-0.3		Min	0.403	0.042	0.000	0.025	0.271	0.283	0.022	0.000	0.025	0.181
		Max	0.514	0.077	0.001	0.035	0.356	0.354	0.057	0.000	0.034	0.232
												
	0.1-0.3	Mean	0.501	0.004	0.004	0.071	0.275	0.357	0.002	0.002	0.056	0.171
		Min	0.448	0.003	0.003	0.065	0.236	0.336	0.001	0.001	0.053	0.161
		Max	0.544	0.006	0.008	0.085	0.318	0.377	0.003	0.003	0.059	0.188
												
	0.3-0.5	Mean	0.505	0.003	0.201	0.090	0.127	0.363	0.002	0.194	0.062	0.086
		Min	0.464	0.002	0.182	0.082	0.117	0.340	0.002	0.168	0.060	0.081
		Max	0.552	0.004	0.221	0.108	0.139	0.389	0.003	0.222	0.065	0.091
												
	< 0.1	Mean	0.476	0.188	0.000	0.051	0.137	0.326	0.182	0.000	0.042	0.095
0.3-0.5		Min	0.424	0.158	0.000	0.042	0.125	0.288	0.152	0.000	0.034	0.083
		Max	0.537	0.209	0.000	0.062	0.150	0.365	0.215	0.000	0.049	0.105
												
	0.1-0.3	Mean	0.506	0.201	0.003	0.090	0.128	0.365	0.194	0.002	0.062	0.086
		Min	0.460	0.170	0.002	0.080	0.116	0.347	0.170	0.002	0.059	0.081
		Max	0.548	0.223	0.005	0.101	0.140	0.388	0.221	0.004	0.065	0.092
												
	0.3-0.5	Mean	0.512	0.200	0.199	0.095	0.101	0.373	0.193	0.193	0.063	0.067
		Min	0.479	0.158	0.178	0.083	0.089	0.350	0.167	0.167	0.061	0.065
		Max	0.559	0.226	0.220	0.107	0.113	0.397	0.220	0.221	0.066	0.071

* Proportion of marker pairs that *X*^2^ exceeded 3.84, the 5% critical value of $\chi_{\left(1\right)}^{2}$.

The power estimates of chi-square tests for digenic, trigenic, and quadrigenic disequilibria increased with the increasing MAF at both loci, whereas those for zygotic LD decreased with the increasing MAF (Table 7). For example, for intra-chromosome pairs, the estimates of power for composite LD increased from 0.389 when MAF at both loci were less than 0.1 to 0.512 when MAF at both loci were above 0.3; in contrast, the power estimates for zygotic LD decreased from around 0.3 when MAF at both loci were below 0.30 to about 0.1 when MAF at either locus was above 0.3. In all nine MAF classes, the power estimates for trigenic and quadrigenic disequilibria were much smaller than those for digenic disequilibria. In particular, the power estimates for trigenic and quadrigenic disequilibria in most MAF classes were below 0.05, confirming the hypothesis of zero trigenic and quadrigenic disequilibria. Patterns of changes in the estimates of power for individual genic disequilibria with gene frequency were similar for intra- and inter-chromosome pairs but the power estimates were generally smaller for inter-chromosome pairs than for intra-chromosome pairs throughout all MAF classes.

## Discussion

This study represents the first major genome-wide survey of high-order genic disequilibria between three or four genes at pairs of loci in a farmed animal species. We chose the Kinsella composite beef population for such a survey because continued crossbreeding and selection for growth and cow reproduction would have made the population to stay in a HWD condition, thereby providing an excellent opportunity for uncovering the high-order genic disequilibria. The survey showed that the trigenic and quadrigenic disequilibria were generally two to three times smaller than the usual digenic disequilibria (gametic or composite LD) (Table 5). Correspondingly, there was less power of testing for these high-order genic disequilibria than for the digenic disequilibria (Tables 2 and 3). The magnitude and power of LD decreased with the distance between markers in close proximity (≤ 5 Mb) though the decay was much more obvious for the digenic disequilibria than for high-order disequilibria (Table 1; Figure 1).

The power estimates for zygotic LD and its components (Tables 2 and 3) were much higher than the expected value of 0.05 for unlinked marker pairs with a distance of >50 Mb. This expectation would be true if physical linkage between SNPs is the only cause of LD. It is well known that LD may arise from many evolutionary and demographic factors such as selection, random drift, inbreeding, mutation and population admixture and such LD can occur between linked as well as independent loci [3,11]. Goddard and Hayes [3] cited the small effective population size (*N*_*e*_) as a major cause of LD in cattle populations. The effective population size was large (>50,000) before domestication, but was drastically declined to 1,000-2,000 after domestication. In many cattle breeds, this number was further declined to approximately 100 after recent breed formation. This sharp decline in *N*_*e*_ causes some LD to exist at long distances. The strong effects of selection and population admixture expected in our crossbred cattle population would also contribute further to greater LD. It should be recognized that the effect of selection on LD involves markers or genes that are localized at certain parts of the genome and thus selection-induced LD would have no or little relation with physical distance.

Most studies have focused on gametic LD for individual pure-breed cattle populations. Our estimates of gametic LD for the Kinsella composite beef population are similar to those reported for Holstein and other pure-breed cattle in the recent literature [4-8]. In fact our estimates are generally slightly lower from different comparisons. Khatkar et al. [4] observed the genome-wide $r_{\mathrm{GLD}}^{2}$ average of 0.016, in comparison to our estimate of 0.0105. Khatkar et al. [4] used a smaller set of markers (15,036 SNPs) covering all 29 autosomes but a larger number of animals (1,546). Sargolzaei et al. [5] presented $r_{\mathrm{GLD}}^{2}$ values between adjacent markers with a genome-wide mean of 0.31, comparing to our mean of 0.195 (detailed data not shown). However, an updated study by the same group [6] with more markers (38,590 SNPs) had a genome-wide $r_{\mathrm{GLD}}^{2}$ average of 0.20 which is very close to our value. Villa-Angulo et al. [7] calculated $r_{\mathrm{GLD}}^{2}$ values using 101 targeted high-density regions (non-overlapping genomic windows of 100 kb containing 10 or more markers and a maximum gap between markers of 20 kb) on QTL-rich chromosomes 6, 14 and 25 to calculate values for 19 beef and dairy breeds; the mean values of $r_{\mathrm{GLD}}^{2}$ ranged from 0.204 for Nelore to 0.397 for Hereford with an overall average of 0.294. Qanbari et al. [8] obtained a genome-wide $r_{\mathrm{GLD}}^{2}$ average of 0.30 for SNP pairs with a distance of < 25 kb for German Holstein, which is very comparable to our estimate of 0.285 for the same distance range (<25 kb) (data not shown). Since our $r_{\mathrm{GLD}}^{2}$ and $r_{\mathrm{CLD}}^{2}$ estimates are very similar, the above comparisons of gametic LD estimates with other studies would be applicable to composite LD estimates as well. However, no comparison on zygotic LD ($r_{\mathrm{CLD}}^{2}$) was possible because other studies have not provided the estimates of zygotic LD. If such estimates of zygotic LD were available, they would have had a similar pattern as in our study. Overall, we conclude that LD useful for genomic selection most likely occurs between those markers that are adjacent or tightly linked.

It is somewhat surprising to see similar values of gametic LD in our crossbred population and in those pure-bred populations as described above. In the pure-breed studies, the focus has been on gametic LD that is estimable often under the HWE assumption [9]. We suggest that the similarity may be due to lack of strong HWD in our beef population. In our study, significant HWD was found at a total of 4,024 (8.2%) SNPs over the genome (Table A 3). This is only slightly higher than 5%, the probability of significant HWD by chance alone. Higher HWD would be expected in our beef population because of continued crossbreeding of animals with diverse breed (genetic) backgrounds every generation. While the data quality control measures such as the removal of ~17% of SNP loci especially with MAF <2.0% and HWD chi-square values of >600 might have contributed to the reduced HWD, it could also be that after several generations of crossbreeding, certain level of genetic homogeneity might have been achieved. In other words, genetic integrity of distinct breeds is no longer clear. Thus, while our gametic LD was estimated without the HWE assumption, the Kinsella composite beef population may be closer to the HWE condition than what we have thought in the past.

To the best of our knowledge, the only other survey of high-order genic disequilibria was made by Liu et al. [13]. Using the essentially same statistical analysis as in our study, Liu et al. [13] observed that the genome-wide percentages of significant composite LD, two trigenic disequilibria and quadrigenic disequilibrium were 61%, 23%, 19%, and 22%, respectively. These power estimates are clearly higher than those observed in our study (Tables 2 and 3). Several factors may contribute to the difference in genic disequilibria and their test power between the two studies. First, In order to fit the simple biallelic model for detecting individual genic disequilibria, Liu et al. [13] used the most frequent allele and a new synthetic allele consisting of all other alleles for the microsatellite markers with more than two alleles observed in [18]. The use of the synthetic allele certainly reduces the likelihood of detecting low MAF. In contrast, our less stringent threshold of MAF ≥ 2% at diallelic SNPs would allow for the presence of low MAF. Second, 148 dogs sampled from the pedigree by Liu et al. [13] were closely inbred relatives and the pedigree was established from a limited number of founders (seven greyhounds and six Labrador retrievers). Thus, strong founder effect coupled with high level of inbreeding would have caused large LD in the dog population whereas our composite beef population with a large number of founders and repeated crossbreeding should have only had a limited founder effect or inbreeding effect on LD. Third, Liu et al. [13] observed a much greater chromosome-to-chromosome variation in individual genic disequilibria than we did in our study. Such inter-chromosome variation is due in part to the difference in marker density between the two studies. The limited number of markers sampled from individual chromosomes across the canine genome would make the study by Liu et al. [13] more likely to suffer from biased sampling of the genome. Fourth, with a small sample size (148 dogs) in Liu et al. [13], the tests for individual genic disequilibria must be based on a two-way contingency table with many empty cells. Due to unpredictable distributions of these empty cells in the two-way table, the genic disequilibria might have been under- or over-emphasized, thereby resulting in a much wider range of the power values.

Our study showed that digenic disequilibria (gametic or composite LD) were much more important than trigenic and quadrigenic disequilibria in terms of magnitudes and test power. This observation coupled with the similarity of the two digenic disequilibria (gametic and composite LD) is consistent with the low level of HWD in our beef population (only 8.2% of SNPs had a significant HWD as shown in Table A3). Under HWE, all non-gametic associations would be zero and thus composite LD would equal to gametic LD and three- and four-gene disequilibrium would be negligible [19,20]. Moreover, in developing the theory underlying the statistical analysis used here, Weir and Cockerham [19] assumed the random union of gametes that would have taken from an infinite large founder population so that all initial disequilibrium would be digenic (gametic LD). It is evident from Table 6.4 of Weir and Cockerham [19] that individual genic disequilibria in subsequent inbred generations decay by a rate gauged in terms of two-locus descent measures. Further numerical results (Table 6.5 of Weir and Cockerham [19]) showed that relative to the digenic disequilibria, the trigenic and quadrigenic disequilibria would be always small in a given inbred generation and they could take a long time to reach the equilibrium values. Thus, it is expected that the digenic LDs overpower the high-order disequilibria.

Our study is the first empirical evaluation of the dependence of LD on allele frequency. The power estimates of chi-square tests for digenic, trigenic and quadrigenic disequilibria increased with the increasing gene frequency at both loci, but those for zygotic LD decreased with the increasing gene frequency. Weir and Cockerham [19] used extensive computer simulations to show the similar trend for individual genic disequilibria but these authors did not consider zygotic LD. The simulation results also confirmed that the allowance for digenic disequilibria not only led to nonzero gametic or composite LD as expected, but also to nonzero quadrigenic disequilibrium as implied by the power of the chi-square test being more than 5%. It is difficult to explain exactly the trend for zygotic LD. Since zygotic LD is a complex function of individual genic disequilibria weighted by gene frequency [11,19], the combinations of gene frequencies and individual disequilibria are too numerous to identify the exact combinations of genic disequilibria at different gene frequencies for the observed trend of zygotic LD. This will certainly be an area for more research in the future.

In our study, we proposed an *ad hoc* measure of non-allelic associations ($\varphi^{2}$) based on chi-square statistics for individual genic disequilibria. It is equal to the squared correlation (*r*^2^) only when the chi-square statistics are calculated from a 2 × 2 contingency table [21]. We used this *ad hoc* measure for estimating the degree of association. More importantly the measure was also used for detecting outlier chi-square statistics by setting the range of the observed $\varphi^{2}$ values as 0 ≤ $\varphi^{2}$ ≤ 1 for individual genic disequilibria. Before the removal of outliers, we observed extremely large standard deviations of chi-square values over marker pairs in many frequency classes for the trigenic and quadrigenic disequilibria. Weir and Cockerham [19] noted in their simulation study a similar problem of unusually large standard deviations of chi-square values but offered no explanation about it. After the removal of outliers, we noted that all standard deviations of chi-square values fell within the normal range. Of course, we used a pragmatic and conservative approach to trim off the outlier chi-square statistics. When the $\varphi^{2}$ value is calculated from a general *I* × *J* contingency table, its allowable range is given by 0 ≤ $\varphi^{2}$ ≤ min[(*I*-1), (*J*-1)] [16]. It is already known (e.g., [12], [20]) that the gametic and zygotic LD are calculated from a 2 × 2 contingency table and thus their acceptable range should be 0 ≤ $\varphi^{2}$ ≤ 1. However, it remains unclear of the size of the contingency tables for other genic disequilibria. Thus, more research is needed to determine appropriate contingency tables for digenic, trigenic, and quadrigenic disequilibria.

Our study has practical implications. The main result from our study is the predominance of digenic disequilibria coupled with insignificant high-order disequilibria. This result supports the current intensive effort of using gametic LD for GWAS and genomic selection in cattle and other animal species. It has been demonstrated (e.g., [3]) that gametic LD in domestic animals may occur at a long distance (> 1 cM) due to the rapid and sudden decline of population size (bottleneck effect) during domestication and at breed formation, in comparison to the situation in human where there is no gametic LD at long distances. However, it has also been suspected that such LD may be in part due to false positive associations resulted from a mixture of multiple breeds or other causes. If the breeds can be identified through pedigree information, Goddard and Hayes [3] suggested the use of breeds as a covariate in the statistical model to minimize such false positive associations. However, animals in our composite beef population had been produced through multi-sire breeding group natural service on pasture over 4-5 generations. Clearly, their breed identity is no longer available despite the inference of parent identity based on the BovineSNP50 Beadchip. So the suggestion by Goddard and Hayes [3] may not be feasible for this composite beef population. Nevertheless, since lack of high-order trigenic and quadrigenic disequilibria coupled with the evidence of insignificant HWD would indicate little non-gametic multilocus correlation [19], our analysis may provide a quick, practical means of assessing the importance of the false positive associations in a multi-breed population.

## Conclusions

This study is the first major genome-wide survey of high-order genic disequilibria between three or four genes at pairs of 50K SNP markers in a farmed animal species. The survey showed that the trigenic and quadrigenic disequilibria were generally insignificant and two- to three-fold smaller than the usual digenic disequilibria (gametic or composite LD). The powers of tests for these high-order genic disequilibria dropped rapidly even at a very short distance between SNPs. These results support the current intensive focus on the use of gametic LD for GWAS and genomic selection activities in the Kinsella composite beef population.

## Methods

### Description of animals and genotyping data

The Kinsella beef composite population has been described in our recent studies on QTL mapping, candidate gene identification and genomic selection [e.g., 14-17]. Here we recapitulate the essential details of this population. It was produced by crossing between Angus, Charolais, or University of Alberta hybrid bulls and a hybrid dam line. The hybrid dam line was obtained by crossing among three composite cattle lines, namely beef synthetic 1 (SY1), beef synthetic 2 (SY2) and dairy × beef synthetic (SD) for more than 10 years after 30 years (1960-1990) single-sire crossbreeding. SY1 was composed of approximately 33% each of Angus and Charolais, 20% Galloway, 5% Brown Swiss, and small amounts of other breeds. SY2 was composed of approximately 60% Hereford and 40% other beef breeds mainly including Augus, Charolais and Galloway. SD was composed of approximately 60% dairy cattle (Holstein, Brown Swiss, or Simmental) and approximately 40% of other breeds, mainly including Angus and Charolais [22]. The blood samples of 1023 beef steers were collected and genotyped using the Illumina Infinium genotyping system with the BovineSNP50 Beadchip. All steers were produced from multi-sire breeding group natural service on pasture. The sire genotype of each calf was determined in a parentage test by using the BovineSNP50 Beadchip, but the parentage of about 100 animals remained unknown because these animals were either sires at initial crossing or sires without progeny. There were 116 sire families with varying family sizes ranging from one to 54 progeny per family. It is estimated that there have been about 4-5 generations since initial crossing.

A total of 51,828 SNP markers were originally obtained in the genotyped data. These markers were distributed across 29 autosomes and one sex chromosome in the entire bovine genome. For our analyses, we only used 43,124 SNPs after removing those markers (i) with monomorphism, (ii) with unknown genomic position and (iii) on the sex chromosome, (iv) with minor allele frequency (MAF) of ≤ 2% [1], and (v) with a Chi-square value >600 for the HWD test.

### Components of zygotic linkage disequilibrium

For two loci, each with two alleles, *A* and *a* at locus *A* and *B* and *b* at locus *B*, there are nine possible genotypes (ten if the coupling and repulsion double heterozygotes are distinguishable). Following Yang [23], we wrote frequencies of these genotypes as, $P_{\mathrm{vz}}^{\mathrm{uy}}=P_{\mathrm{uy}}^{\mathrm{vz}}$, which result from union of gametes *uy* and *vz* with *uv* = *A* or *a*, and *yz* = *B* or *b*. The genotypic frequencies at individual loci are the marginal totals of the appropriate two-locus genotypic frequencies. For example, the frequency of genotype *AA* is,

(1) $$P_{A\cdot }^{A\cdot }=P_{\mathrm{AB}}^{\mathrm{AB}}+P_{\mathrm{Ab}}^{\mathrm{AB}}+P_{\mathrm{Ab}}^{\mathrm{Ab}}\text{.}$$

With the genotypic frequencies at locus *A*, the frequency of allele *A* is, pA=PA⋅A⋅+12Pa⋅A⋅ and that of allele *a* is *p*_*a*_ = 1- *p*_*A*_.

Departures from HWE at locus *A* are, DA=PA⋅A⋅−pA2=Pa⋅a⋅−pa2=−12Pa⋅A⋅−pApa and those at locus *B* are, DB=P⋅B⋅B−pB2=P⋅b⋅b−pb2=−12P⋅b⋅B−pBpb.

In a random mating population, HWD disappears (i.e., *D*_*A*_ = *D*_*B*_ = 0). In a non-random mating population, nonzero HWD is measured by the fixation index which can be either positive when there is inbreeding or negative when inbreeding is avoided. For example, the HWD at locus *A* can be written as *D*_*A*_ = *f*_*A*_*p*_*A*_*p*_*a*_, where $f_{A}=\frac{P_{A\cdot }^{A\cdot }-p_{A}^{2}}{p_{A}p_{a}}=\frac{P_{a\cdot }^{a\cdot }-p_{a}^{2}}{p_{A}p_{a}}=1-\frac{P_{a\cdot }^{A\cdot }}{2p_{A}p_{a}}$, is the fixation index at locus *A*, with -1 ≤ *f*_*A*_ ≤ +1.

It was established [11,12] that the total zygotic LD between loci *A* and *B* could be defined in terms of zygotic LDs for individual genotypes with each zygotic LD being a complex function of digenic, trigenic and quadrigenic disequilibria. For example, the zygotic LD for double homozygote *AABB* ($\omega_{\mathrm{AB}}^{\mathrm{AB}}$) would simply be the deviation of the frequency of double homozygote from the product of the corresponding homozygotes at loci *A* and *B*

(2) $$\omega_{\mathrm{AB}}^{\mathrm{AB}}=P_{\mathrm{AB}}^{\mathrm{AB}}-P_{A\cdot }^{A\cdot }P_{\cdot B}^{\cdot B}=2p_{A}D_{.B}^{\mathrm{AB}}+2p_{B}D_{A.}^{\mathrm{AB}}+2p_{A}p_{B}D_{..}^{\mathrm{AB}}+2p_{A}p_{B}D_{.B}^{A.}+{\left(D_{..}^{\mathrm{AB}}\right)}^{2}+{\left(D_{.B}^{A.}\right)}^{2}+D_{\mathrm{AB}}^{\mathrm{AB}}$$

where each genic disequilibrium (*D*) is the deviation of a frequency from that based on random association of genes and accounting for any lower order disequilibria. The usual gametic LD ($D_{..}^{\mathrm{AB}}$) would be the deviation of frequency of gamete *AB* from the product of frequencies of allele *A* at locus *A* and allele *B* at locus *B*$D_{..}^{\mathrm{AB}}=P_{..}^{\mathrm{AB}}-p_{A}p_{B}$ with $P_{..}^{\mathrm{AB}}=P_{\mathrm{AB}}^{\mathrm{AB}}+P_{\mathrm{Ab}}^{\mathrm{AB}}+P_{\mathrm{aB}}^{\mathrm{AB}}+P_{\mathrm{ab}}^{\mathrm{AB}}$.

When zygotes arise from random union of gametes as often assumed in most LD studies, all non-gametic disequilibria including HWD would disappear (*e.g.*, $D_{A.}^{A.}=D_{.B}^{A.}=D_{.B}^{\mathrm{AB}}=D_{\mathrm{AB}}^{\mathrm{AB}}=0$). In this case, the zygotic LD for genotype *AABB* ($\omega_{\mathrm{AB}}^{\mathrm{AB}}$) would reduce to, $\omega_{\mathrm{AB}}^{\mathrm{AB}}=2p_{A}p_{B}D_{..}^{\mathrm{AB}}+{\left(D_{..}^{\mathrm{AB}}\right)}^{2}$.

This formula is the basis for possible use of double homozygosity to measure gametic LD in a random mating population [24,25].

Since the two types of double heterozygote (*AB*/*ab* and *Ab*/*aB*) in our unphased SNP data could not be distinguished, we used the composite LD (Δ_*AB*_) and a composite quadrigenic component (Δ_*AABB*_) in place of gametic and quadrigenic disequilibria. Thus, the zygotic LD for genotype *AABB* ($\omega_{\mathrm{AB}}^{\mathrm{AB}}$) in equation (1) was rewritten as

(3) $$\omega_{\mathrm{AB}}^{\mathrm{AB}}=P_{\mathrm{AB}}^{\mathrm{AB}}-P_{A\cdot }^{A\cdot }P_{\cdot B}^{\cdot B}=2p_{A}D_{\mathrm{ABB}}+2p_{B}D_{\mathrm{AAB}}+2p_{A}p_{B}\Delta_{\mathrm{AB}}+\Delta_{\mathrm{AB}}^{2}+\Delta_{\mathrm{AABB}}$$

where

(4) $$\Delta_{\mathrm{AB}}=P_{\cdot \cdot }^{\mathrm{AB}}+P_{\cdot B}^{A\cdot }-2p_{A}p_{B}=D_{\cdot \cdot }^{\mathrm{AB}}+D_{\cdot B}^{A\cdot }$$

and

(5) $$\Delta_{\mathrm{AABB}}=D_{\mathrm{AB}}^{\mathrm{AB}}-2D_{\cdot \cdot }^{\mathrm{AB}}D_{\cdot B}^{A\cdot }$$

It should be noted from equations (1) and (2) that the two trigenic disequilibria in (2) were rewritten without superscripts for notational simplicity.

### Maximum likelihood estimation

Following Weir and Cockerham [19] and Weir [20], we used the procedure of statistical inference based on the assumption of multinomial sampling of individual diploids from a population. The observed frequencies and disequilibria with tildes (~) were maximum likelihood (ML) estimates of corresponding parametric values. Since the additive models described earlier allowed for defining the same number of parameters as there would be degrees of freedom, the ML estimates were simply replacing all parametric values of frequencies and disequilibria with corresponding observed values. For example, the ML estimates of composite LD were simply given by, $\begin{array}{lll} {\tilde{\Delta }}_{\mathrm{AB}} & = & {\tilde{P}}_{\cdot \cdot }^{\mathrm{AB}}+{\tilde{P}}_{\cdot B}^{A\cdot }-2{\tilde{p}}_{A}{\tilde{p}}_{B}\\ & = & {\tilde{D}}_{\cdot \cdot }^{\mathrm{AB}}+{\tilde{D}}_{\cdot B}^{A\cdot } \end{array}$

However, the ML estimates might be biased because they would involve quadratic terms of multinomial variables. For example, the expectation of the squared gene frequency of allele *A* over replicate samples of size *n* would be, $E({\tilde{p}}_{A}^{2})=p_{A}^{2}+[p_{A}(1-p_{A})+D_{A}]/2n$

where *D*_*A*_ is the HWD measure at locus *A*[20]. With the sufficiently large sample (*n* = 1023 animals) in our data set, we invoked large-sample theory for statistical inference about genic disequilibria. Thus, we ignored the possible biases of order 1/*n*.

### Hypothesis testing and power

With a ML estimate ($\tilde{D}$ or $\tilde{\Delta }$) of a given genic disequilibrium *D* or *Δ*, along with its sampling variance, [Var($\tilde{D}$) or Var($\tilde{\Delta }$)] being given in Appendix in the Additional file 1 section, we constructed a test statistic, $X^{2}={\tilde{D}}^{2}/Var(\tilde{D})$ or $X^{2}={\tilde{\Delta }}^{2}/Var(\tilde{\Delta })$

to test the hypothesis of zero disequilibrium (i.e., H_0_: *D* = 0 or H_0_: *Δ* = 0). Assuming the asymptotic normality of the ML estimate, *X*^2^ under the hypothesis of zero disequilibrium would be distributed as chi-square with one degree of freedom.

As usual, each chi-square test would commit two kinds of error: a true hypothesis may be rejected (Type I error) or a false hypothesis may not be rejected (Type II error). The probability of Type I error is measured by the significance level whereas the probability of Type II error is often related to the power of the test. Generally, as the power is the probability of rejecting a false hypothesis, it equals to one minus the probability of Type II error. In the present study, however, we adopted a different use of the power as proposed by Weir and Cockerham ([19], p. 100) and Weir ([20], p. 110): we calculated the power when the hypothesis being tested is true. In this particular case, a power value equals to the significance level.

### Chi-square statistic and correlation

In the past, the squared correlation (*r*^2^) has been routinely used as a measure of gametic LD ($r_{\mathrm{GLD}}^{2}$), composite LD ($r_{\mathrm{CLD}}^{2}$), or zygotic LD ($r_{\mathrm{ZLD}}^{2}$). We used a chi-square statistic ($X_{i}^{2}=nr_{i}^{2}$*i* = *GLDCLD* or *ZLG*) to test for the significance of the LD estimate. It is known from the literature [21] that the relationship of$X_{i}^{2}=nr_{i}^{2}$ would hold exactly only for a 2 × 2 contingency table. This was the case for GLD and ZLD, but not for CLD. When dropping out three- and four-gene disequilibria in testing for zero composite LD (${\tilde{\Delta }}_{\mathrm{AB}}=0$), we would obtain an approximate chi-square statistic, $X_{\mathrm{AB}}^{2}\approx n{\tilde{\Delta }}_{AB}^{2}/[({\tilde{\pi }}_{A}+{\tilde{D}}_{A})({\tilde{\pi }}_{B}+{\tilde{D}}_{B})]$.

Which would be equal to $nr_{\mathrm{CLD}}^{2}$ as given in Weir [26]. Similar approximations or restrictions would be needed if the relationship of$X_{i}^{2}=nr_{i}^{2}$ were desired for three- and four-gene disequilibria. Thus, to avoid such approximations or restrictions, we used a generalized measure of square correlation $\varphi^{2}=X^{2}/n$[21] in place of *r*^2^ as a standardized measure of genic disequilibria. As pointed out above, the relationship of $\varphi^{2}=r^{2}$ would hold only for a 2 × 2 contingency table.

### Data analysis

All data analysis and required computation were done using SAS 9.3 [27]. The calculation of gametic and composite LD was carried out using PROC ALLELE of SAS/Genetics 9.3. In this calculation, the SNP marker data was read in as columns of genotypes using the GENOCOL and DELIMITER= options in the PROC ALLELE statement; gametic LD was calculated if the HAPLO= EST option in the PROC ALLELE statement was invoked, whereas composite LD was calculated if the HAPLO= NONEHWD option was specified. Zygotic LD and its components as well as hypothesis testing were calculated using SAS Macro language and SAS/IML procedure.

## Abbreviations

bp, base pairs; cM, Centimorgan; CLD, Composite linkage disequilibrium; GLD, Gametic linkage disequilibrium; GWAS, Genome-wide association studies; HWD, Hardy-Weinberg disequilibrium; HWE, Hardy-Weinberg equilibrium; kp, Kilo base pairs; LD, Linkage disequilibrium; MAF, Minor allele frequency; Mb, Mega base pairs; QTN, Quantitative trait nucleotide; SNP, Single nucleotide polymorphism; ZLD, Zygotic linkage disequilibrium.

## Competing interests

The authors declare that they have no competing interests.

## Authors' contributions

This research represents a portion of a thesis submitted by QJ to the University of Alberta in partial fulfilment of the requirements for the Master of Science degree. QJ, ZW and R-CY designed the study. QJ and R-CY analyzed the data and wrote the manuscript. ZW edited the manuscript. SSM provided intellectual inputs to the study. All authors read and approved the final manuscript.

## Supplementary Material

Additional file 1 **Table A1.** Number of Single Nucleotide Polymorphism (SNP) markers (m) and chromosome length (mega base pairs, Mb) for 29 bovine autosomes (BTA 1 to BTA 29) in the Kinsella composite beef population. Mean, standard deviation (SD), minimum and maximum distances (in base pairs) between all pairs of adjacent markers are also presented. **Table A2.** The proportion of syntenic SNP pairs with out-of-bound estimates of generalized measures of squared correlation for trigenic and quadrigenic disequilibria in the Kinsella composite population. **Table A3.** Summary statistics on single-locus heterozygosity, fixation index and Hardy-Weinberg disequilibrium (HWD) averaged over all SNP markers on 29 bovine autosomes (BTA 1 to BTA 29) in the Kinsella composite beef population. **Appendix:** Sampling variances of individual genic disequilibria in zygotic LD. Click here for file
