# Supplementary material for: Genome-wide analysis of zygotic linkage disequilibrium and its components in crossbred cattle
Source: BMC Genet. 2012 Jul 24;13:65. doi: 10.1186/1471-2156-13-65 (PMC3443453; doi:10.1186/1471-2156-13-65)
Supplement: Additional file 1 — Table A1. Number of Single Nucleotide Polymorphism (SNP) markers (m) and chromosome length (mega base pairs, Mb) for 29 bovine autosomes (BTA 1 to BTA 29) in the Kinsella composite beef population. Mean, standard deviation (SD), minimum and maximum distances (in base pairs) between all pairs of adjacent markers are also presented. Table A2. The proportion of syntenic SNP pairs with out-of-bound estimates of generalized measures of squared correlation for trigenic and quadrigenic disequilibria in the Kinsella composite population. Table A3. Summary statistics on single-locus heterozygosity, fixation index and Hardy-Weinberg disequilibrium (HWD) averaged over all SNP markers on 29 bovine autosomes (BTA 1 to BTA 29) in the Kinsella composite beef population. Appendix: Sampling variances of individual genic disequilibria in zygotic LD. [file 1471-2156-13-65-S1.pdf]

Additional files

Table A1 Number of Single Nucleotide Polymorphism (SNP) markers (m) and chromosome length (mega base pairs, Mb) for 29 bovine autosomes (BTA 1 to BTA 29) in the Kinsella composite beef population. Mean, standard deviation (SD), minimum and maximum distances (in base pairs) between all pairs of adjacent markers are also presented.

BTA	SNPs (m)	Length (Mb)	Adjacent Marker Distance (bp)			
			Mean	SD	Minimum	Maximum
1	2841	161	56658	46568	131	470043
2	2320	141	60578	59780	75	661839
3	2180	128	58700	57515	108	807545
4	2126	124	58388	47620	3406	430390
5	1815	126	69347	73232	2444	1115633
6	2183	123	56112	51997	2660	826193
7	1895	112	58974	55986	3	950817
8	2026	117	57742	47799	3416	489257
9	1747	108	61883	57633	449	729114
10	1844	106	57363	68139	284	2081464
11	1892	110	58194	52300	886	890683
12	1393	85	61205	60291	237	760907
13	1491	84	56427	47106	382	592177
14	1440	81	56479	45280	108	575964
15	1400	85	60446	54116	3701	683257
16	1326	78	58774	59748	178	1051359
17	1331	77	57408	49438	333	725528
18	1109	66	59597	57330	5603	867228
19	1130	65	57604	45262	3937	553067
20	1321	76	57195	50727	9201	837059
21	1129	69	61297	56769	903	742465
22	1061	62	58135	42967	2432	360641
23	900	52	59256	50555	1568	476317
24	1073	65	60570	51851	95	531092
25	813	44	53478	42015	1342	350281
26	884	52	57759	41705	281	373781
27	811	49	60128	78931	151	1889396
28	785	46	58642	47409	675	363454
29	858	52	60327	58828	1860	806694
Overall	43124	2544	58933	54650	3	2081464

Table A2. The proportion of syntenic SNP pairs with out-of-bound estimates of generalized measures of squared correlation for trigenic and quadrigenic disequilibria in the Kinsella composite population.

BTA	$\phi^2_{D_{ABB}}$ [a]		$\phi^2_{D_{AAB}}$ [a]		$\phi^2_{\Delta_{AABB}}$ [a]	
	< 0	>1	< 0	>1	< 0	>1
1	70.18	0.019	70.34	0.017	0	0.001
2	70.47	0.018	69.16	0.016	0	0.001
3	69.12	0.012	68.90	0.013	0	0.001
4	68.51	0.018	67.22	0.016	0	0.001
5	70.75	0.016	69.00	0.018	0	0.002
6	66.62	0.015	66.81	0.016	0	0.001
7	66.68	0.019	68.32	0.015	0	0.002
8	70.32	0.012	69.64	0.016	0	0.001
9	70.42	0.017	70.16	0.014	0	0.001
10	70.32	0.013	70.01	0.011	0	0.002
11	69.00	0.020	69.38	0.021	0	0.001
12	67.45	0.018	67.96	0.018	0	0.001
13	69.69	0.014	67.33	0.016	0	0.003
14	64.77	0.014	66.79	0.018	0	0.001
15	67.48	0.018	69.55	0.019	0	0.002
16	73.03	0.020	71.29	0.022	0	0.001
17	69.73	0.019	68.11	0.017	0	0.001
18	68.56	0.023	69.22	0.016	0	0.001
19	68.02	0.014	66.28	0.016	0	0.001
20	68.28	0.017	69.99	0.019	0	0.001
21	66.70	0.019	66.55	0.014	0	0.002
22	70.78	0.013	73.45	0.014	0	0.002
23	67.99	0.028	70.61	0.014	0	0.000
24	68.40	0.020	67.50	0.020	0	0.002
25	66.58	0.027	64.77	0.020	0	0.002
26	68.87	0.014	68.61	0.011	0	0.002
27	69.31	0.017	69.51	0.022	0	0.002
28	72.49	0.013	73.72	0.019	0	0.001
29	70.16	0.019	68.31	0.018	0	0.002
Overall	68.99	0.020	68.91	0.020	0	0.001

[a] The generalized measures of squared correlation for trigenic and quadrigenic disequilibria: $\phi^2_{D_{ABB}} = X^2_{D_{ABB}}/n$, $\phi^2_{D_{AAB}} = X^2_{D_{AAB}}/n$ and $\phi^2_{\Delta_{AABB}} = X^2_{\Delta_{AABB}}/n$, where n is the number of animals at individual SNP pairs.

Table A3. Summary statistics on single-locus heterozygosity, fixation index and Hardy-Weinberg disequilibrium (HWD) averaged over all SNP markers on 29 bovine autosomes (BTA 1 to BTA 29) in the Kinsella composite beef population.

	Heterozygosity			Fixation index			HWD	
BTA	Mean±SD	Minimum	Maximum	Mean	Minimum	Maximum	# SNPs	% SNPs
1	0.346±0.141	0.038	0.562	-0.004	-0.129	0.41	240	7.5
2	0.359±0.136	0.042	0.563	-0.010	-0.167	0.254	241	9.0
3	0.356±0.136	0.027	0.559	-0.014	-0.144	0.573	229	9.4
4	0.355±0.136	0.038	0.549	-0.006	-0.148	0.44	201	8.3
5	0.356±0.139	0.038	0.554	-0.010	-0.128	0.224	191	9.2
6	0.359±0.138	0.04	0.555	-0.014	-0.148	0.296	239	9.9
7	0.355±0.139	0.039	0.549	-0.010	-0.132	0.242	189	8.7
8	0.354±0.135	0.037	0.544	-0.007	-0.124	0.328	176	7.7
9	0.349±0.140	0.037	0.551	-0.009	-0.108	0.157	133	6.8
10	0.355±0.135	0.039	0.561	-0.005	-0.124	0.322	166	8.0
11	0.354±0.138	0.041	0.599	-0.008	-0.202	0.206	158	7.3
12	0.353±0.138	0.038	0.554	-0.006	-0.122	0.472	114	7.1
13	0.363±0.136	0.04	0.553	-0.012	-0.161	0.191	167	9.7
14	0.366±0.132	0.039	0.546	-0.007	-0.103	0.397	124	7.5
15	0.357±0.134	0.015	0.56	-0.008	-0.128	0.627	153	9.4
16	0.352±0.138	0.038	0.55	-0.006	-0.113	0.442	117	7.7
17	0.351±0.134	0.026	0.544	-0.004	-0.127	0.327	100	6.7
18	0.370±0.131	0.04	0.559	-0.008	-0.142	0.302	93	7.2
19	0.368±0.133	0.041	0.569	-0.005	-0.154	0.238	82	6.3
20	0.355±0.141	0.04	0.561	-0.010	-0.157	0.273	122	8.4
21	0.358±0.136	0.041	0.558	-0.012	-0.124	0.367	111	8.3
22	0.353±0.134	0.04	0.542	-0.005	-0.112	0.176	87	7.0
23	0.374±0.129	0.041	0.553	-0.011	-0.133	0.247	90	8.8
24	0.367±0.134	0.038	0.552	-0.013	-0.127	0.184	119	9.8
25	0.379±0.127	0.041	0.551	-0.003	-0.106	0.188	60	6.4
26	0.352±0.135	0.04	0.543	-0.012	-0.117	0.715	86	8.5
27	0.358±0.134	0.04	0.557	-0.008	-0.115	0.34	76	8.3
28	0.366±0.131	0.04	0.543	-0.009	-0.14	0.198	82	9.3
29	0.363±0.133	0.04	0.569	-0.007	-0.143	0.157	78	7.9
Overall	0.357±0.136	0.015	0.599	-0.008	-0.202	0.715	4024	8.2

Appendix: Sampling variances of individual genic disequilibria in zygotic LD

Weir and Cockerham [19] provided the formulas for the large-sample variances of different components of zygotic LD. These formulas are reproduced here, with minor modifications, for easier reference. Definitions and notations of different genic disequilibria were detailed in the Materials and Methods section.

Gametic disequilibrium $\left(\widetilde{D}_{AB}\right)$

$$\mathrm{Var}\left(\widetilde{D}_{AB}\right) = \left[\pi_A\pi_B + \tau_A\tau_B D_{AB} + D_A D_B - D_{AB}^2 + D_{A/B}^2 + D_{AB}^{AB}\right]/2n.$$

Non-gametic disequilibrium $\left(\widetilde{D}_{A/B}\right)$

$$\mathrm{Var}\left(\widetilde{D}_{A/B}\right) = \left[\pi_A\pi_B + \tau_A\tau_B D_{A/B} + D_A D_B + D_{AB}^2 - D_{A/B}^2 + D_{AB}^{AB}\right]/2n.$$

Composite disequilibrium $\left(\tilde{\Delta}_{AB}\right)$

$$\mathrm{Var}\left(\tilde{\Delta}_{AB}\right) = [(\pi_A + D_A)(\pi_B + D_B) + \tau_A\tau_B\Delta_{AB}/2 + \tau_A D_{ABB} + \tau_B D_{AAB} + \Delta_{AABB}]/n.$$

Trigenic disequilibria $\left(\widetilde{D}_{AAB} \text{ and } \widetilde{D}_{ABB}\right)$

$$\begin{aligned}\mathrm{Var}\left(\widetilde{D}_{AAB}\right) = \{&(\pi_A^2 + \tau_A^2 D_A - D_A^2)(\pi_B + D_B) + \pi_A\tau_A\tau_B\Delta_{AB} \\ &+(1 - 5\pi_A + D_A)\Delta_{AB}^2 + 2\pi_A\tau_A D_{ABB} \\ &+(\tau_A^2\tau_B - 2D_A\tau_B - 4\tau_A\Delta_{AB})D_{AAB} - 2D_{AAB}^2 \\ &+(\tau_A^2 - 2D_A)\left(D_{AB}^{AB} - 2D_{AB}D_{A/B}\right)\}/2n.\end{aligned}$$

$$\begin{aligned}\mathrm{Var}\left(\widetilde{D}_{ABB}\right) = \{&(\pi_B^2 + \tau_B^2 D_B - D_B^2)(\pi_A + D_A) + \pi_B\tau_B\tau_A\Delta_{AB} \\ &+(1 - 5\pi_B + D_B)\Delta_{AB}^2 + 2\pi_B\tau_B D_{AAB} \\ &+(\tau_B^2\tau_A - 2D_B\tau_A - 4\tau_B\Delta_{AB})D_{ABB} - 2D_{ABB}^2 \\ &+(\tau_B^2 - 2D_B)\left(D_{AB}^{AB} - 2D_{AB}D_{A/B}\right)\}/2n.\end{aligned}$$

Quadrigenic disequilibrium $\left(\tilde{\Delta}_{AABB}\right)$

$$\mathrm{Var}\left(\tilde{\Delta}_{AABB}\right) = \{(\pi_A^2 + \tau_A^2 D_A - D_A^2)(\pi_B^2 + \tau_B^2 D_B - D_B^2)$$

$$+2\tau_A\tau_B(\pi_A\pi_B - 4D_AD_B)\Delta_{AB}$$

$$+(\tau_A^2\tau_B^2 - 4\tau_B^2 D_A - 4\tau_A^2 D_B + 4D_AD_B + 2D_A + 2D_B)\Delta_{AB}^2$$

$$-6\tau_A\tau_B\Delta_{AB}^3 + 3\Delta_{AB}^4$$

$$+[2\pi_B\tau_B(\tau_A^2 - 2D_A) + 4\Delta_{AB}(2\tau_A D_B - 2\tau_A\pi_B + \tau_B\Delta_{AB})]D_{AAB}$$

$$+[2\pi_A\tau_A(\tau_B^2 - 2D_B) + 4\Delta_{AB}(2\tau_B D_A - 2\tau_B\pi_A + \tau_A\Delta_{AB})]D_{ABB}$$

$$+2(3D_B - \pi_B)D_{AAB}^2 + 2(3D_A - \pi_A)D_{ABB}^2 + 20\Delta_{AB}D_{AAB}D_{ABB}$$

$$+[(\tau_A^2 - 2D_A)(\tau_B^2 - 2D_B) - 8\tau_A\tau_B\Delta_{AB} + 6\Delta_{AB}^2$$

$$-4\tau_A D_{ABB} - 4\tau_B D_{AAB}]\Delta_{AABB} - \Delta_{AABB}^2\}/n.$$

In these formulas, we make use of the following notation,

$\pi_x = p_x(1 - p_x)$

$\tau_x = 1 - 2p_x$

where p_x is the frequency of allele x.
